# ARCS/AUCS: A rare clinicoradiological presentation

**DOI:** 10.4103/0971-3026.37049

**Published:** 2008-02

**Authors:** Pratish George, Uttam George, Basant Pawar

**Affiliations:** Department of Internal Medicine, Christian Medical College and Hospital, Ludhiana - 141 008, Punjab, India; 1 Department of Radiology, Christian Medical College and Hospital, Ludhiana - 141 008, Punjab, India; 2 Department of Nephrology, Christian Medical College and Hospital, Ludhiana - 141 008, Punjab, India

**Keywords:** ARCS, AUCS, genitourinary anomalies, magnetic resonance imaging

A rare presentation of ARCS (azoospermia, renal anomalies, cervicothoracic spine dysplasia)/AUCS (azospermia, urogenital anomaly, cervicothoracic spine dysplasia) in a patient of end-stage renal disease is described. ARCS/AUCS is analogous to MURCS (Mullerian duct aplasia, renal aplasia, cervicothoracic somite dysplasia) in females.[1,2]

## Case Report

A 27-year-old man was admitted with vomiting, generalized weakness, anorexia, and weight loss. He had a short neck and stature which, however, did not in any way limit his movements or daily activities. No muscular weakness, paresthesia, or bowel or bladder dysfunction was present. He had an uneventful antenatal, birth, and developmental history. There was no significant family history or of any relevant past illness. Development of secondary sexual features was normal and no erectile dysfunction was noticed. However, the volume of his ejaculate was consistently minimal.

**Figure 1:**
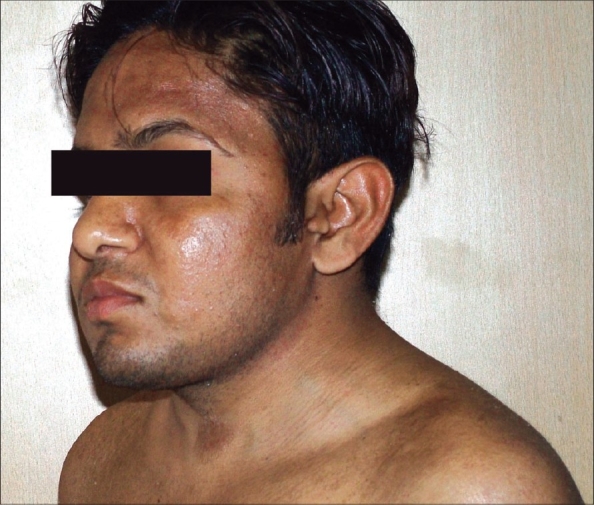
Profile of the patient shows short neck and left preauricular tag

On examination, his height was 162.5 cm and he had a short neck with webbing and Sprengel's deformity. A low hairline with a left auricular tag was seen [Figure 1]. Lateral bending neck movements were impaired. He was pale and had a blood pressure of 190/110 mmHg. Neurological examination revealed bilateral hearing loss, brisk deep tendon reflexes, and flexor plantar response. Cardiovascular and abdominal examinations were normal. Secondary sexual characteristics were normal, but the testes were soft and mildly reduced in size.

**Figure 2:**
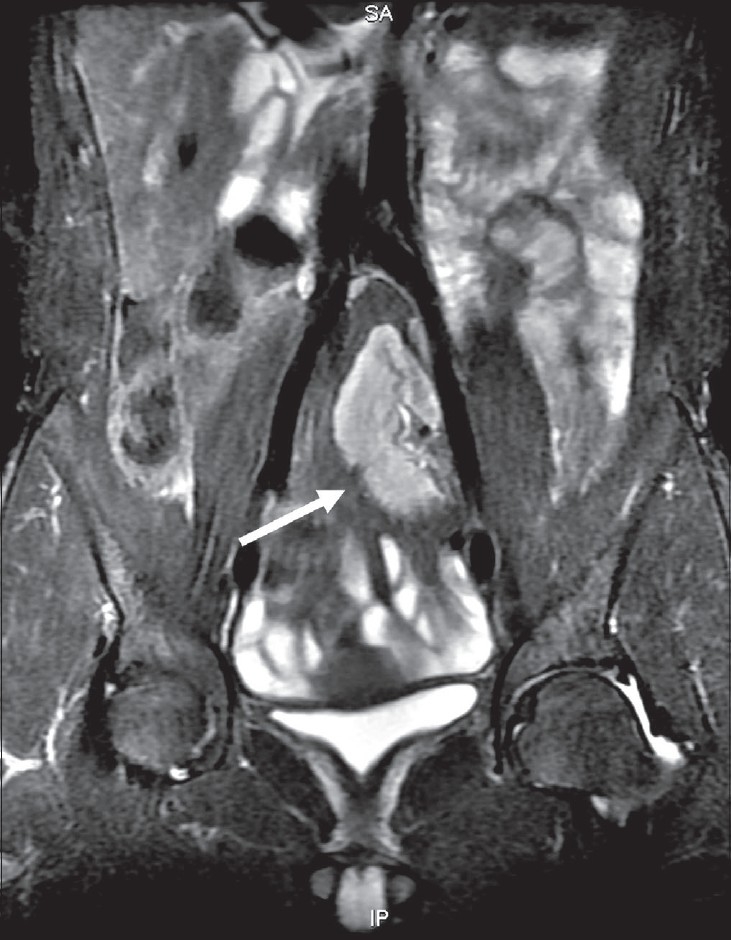
MRI of the lower abdomen and pelvis (coronal fat-suppressed image) shows an ectopic pelvic location of the kidney (arrow). The prostate gland is not visualized

**Figure 3:**
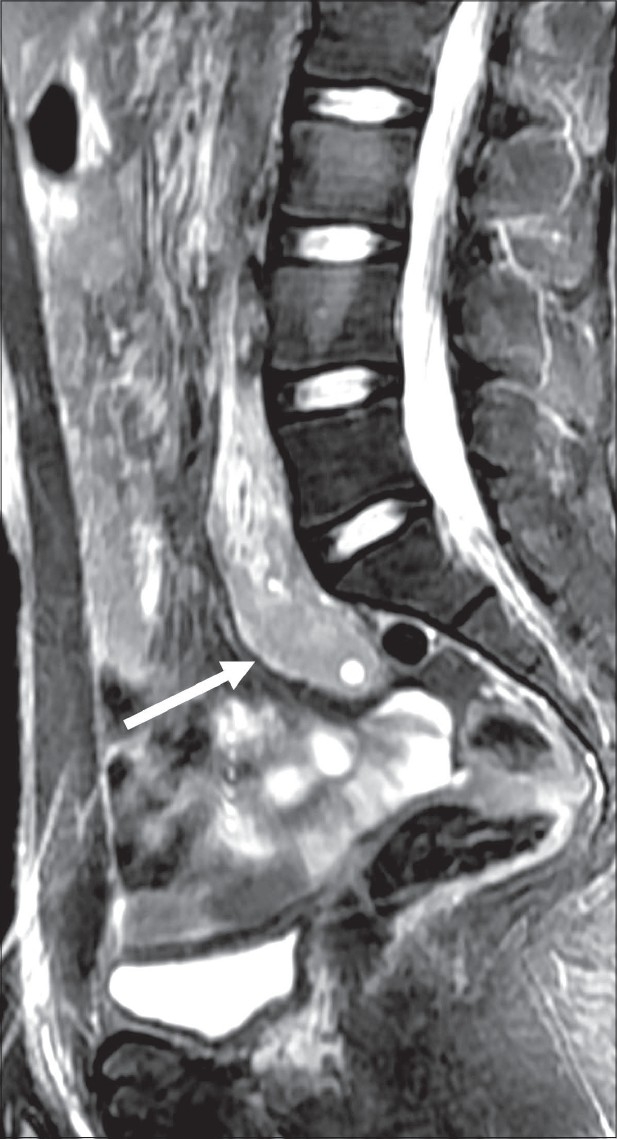
MRI of the lower abdomen and pelvis (sagittal fat-suppressed image) shows an ectopic pelvic location of the kidney (arrow). The prostate gland is not visualized

**Figure 4:**
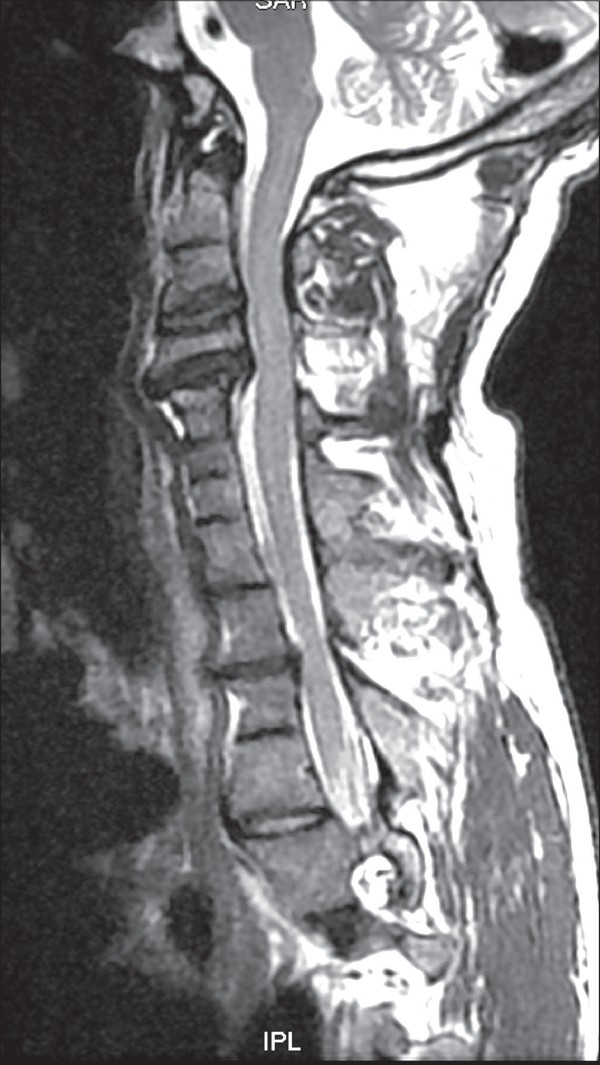
T2W sagittal MRI of the cervical spine shows atlantooccipital fusion and fusion of the C4-C6 vertebrae. The C2-C3 and C3-C4 intervertebral discs show posterior bulges

Investigations showed chronic kidney disease, with anemia and severe metabolic acidosis. The kidneys were not visualized on USG, but a single ectopic kidney was seen in the pelvis on subsequent MRI, anterior to the lower lumbar and upper sacral spine [Figure 2]. The prostate gland and bilateral seminal vesicles were not visualized on transrectal ultrasound (TRUS) and MRI [Figure 3]. Right and left testicular volumes were 9.7 and 7.7 cm^3^, respectively. MRI of the cervical spine showed atlanto-occipital assimilation, fusion of the C4-C6 vertebrae, with intervertebral disc herniation and spinal cord indentation at the C2-C3 and C3-C4 levels [Figure 4]. No spinal cord edema was noted. Audiometry showed mild conductive hearing loss. Echocardiography was normal. Testosterone, follicular stimulating hormone, and luteinizing hormone levels, and karyotyping and testicular biopsy were not done at the patient's request.

The patient's spectrum of presenting symptoms, with type II Klippel-Feil (KF) anomaly, conductive hearing loss, renal dysgenesis with end-stage renal disease, and non-visualized prostate and seminal vesicles, was suggestive of a possible MURCS association in a male. A semen analysis to confirm this association showed azoospermia, in one milliliter of ejaculate. ARCS/AUCS was diagnosed. Spinal fusion surgery for stabilization of the cervical spine was advised but refused. He was discharged with a word of caution regarding the need to avoid strenuous activities, trauma, and weight bearing on the neck, and is currently doing well on maintenance hemodialysis.

## Discussion

MURCS association is a rare developmental disorder usually seen in female children. It is characterized by congenital aplasia of the uterus and upper vagina, with normal secondary sexual features and karyotype.[3] There are renal (agenesis, ectopia, or horseshoe kidneys), skeletal (KF spine anomaly, scoliosis, or fused vertebrae), and hearing defects. Rarely cardiac and digital anomalies are present.[4] MURCS in males was first described by Wellesly *et al*. in a patient with KF deformity and unilateral renal agenesis and a hypothesis of Wolffian duct hypoplasia was proposed for the patient's presentation with azoospermia, absent seminal vesicles, and thin vasa deferentia.[5] Thereafter, a few case reports have described MURCS in males, characterized by nonobstructive azoospermia in place of the uterine malformations seen in women. ARCS[1] and AUCS[2] are terms that have been used to describe this association in males.

The Mullerian and Wolffian ducts are the primordia for the development of the female and male reproductive organs, respectively. They coexist in the undifferentiated embryo until differentiation of the genetic sex. The male testicular production of anti-Mullerian hormones and androgens leads to maturation of the Wolffian duct into the vas deferens and seminal vesicles.[6] The blastema of the cervical vertebrae, scapulae, and the genitourinary system have an intimate spatial relationship at the end of the fourth or beginning of the fifth week of fetal life. An alteration in this region can thus cause cervical vertebrae, scapular, and genitourologic changes.[7] Renal abnormalities in KF and ARCS/AUCS association can be explained on this basis. The cause of the triad of involvement however remains elusive.[1] Genetic studies are unavailable, probably because of the rarity of the syndrome. The chromosomal study has been reported as normal in one patient.[5]

Wolffian defects and ARCS/AUCS should be considered in men presenting with KF anomalies. USG evaluation of the genitourinary system should be done.[8] Rarely, as in this patient, MRI is useful in visualizing the genitourinary system, especially when USG is inconclusive and intravenous urography or CT scan are unsafe or unlikely to be informative because of poor renal function.
